# Medical Service Utilization and Direct Medical Cost of Stroke in Urban China

**DOI:** 10.34172/ijhpm.2020.111

**Published:** 2020-07-20

**Authors:** Dawei Zhu, Xuefeng Shi, Stephen Nicholas, Siyuan Chen, Ruoxi Ding, Lieyu Huang, Yong Ma, Ping He

**Affiliations:** ^1^China Center for Health Development Studies, Peking University, Beijing, China.; ^2^School of Management, Beijing University of Chinese Medicine, Beijing, China.; ^3^National Institute of Chinese Medicine Development and Strategy, Beijing University of Chinese Medicine, Beijing, China.; ^4^School of Economics and School of Management, Tianjin Normal University, Tianjin, China.; ^5^Australian National Institute of Management and Commerce, Sydney, NSW, Australia.; ^6^Research Institute for International Strategies, Guangdong University of Foreign Studies, Guangzhou, China.; ^7^Newcastle Business School, University of Newcastle, Newcastle, NSW, Australia.; ^8^School of Public Health, Peking University, Beijing, China.; ^9^Institute of Population Research, Peking University, Beijing, China.; ^10^China Health Insurance Research Association, Beijing, China.

**Keywords:** Stroke, Medical Service Utilization, Direct Medical Cost, Urban China, Cost of Illness, Treatment Cost

## Abstract

**Background:** Understanding the treatment costs of stroke can guide health policies and interventions. However, few studies have analyzed the treatment costs of stroke in China. The aim of this study is to assess stroke-related medical service utilization, direct costs of stroke and associated stroke predictors, and, second, to understand the structure of medical resource use.

**Methods:** This study used a 5% random sample of claim data from China’s Urban Basic Medical Insurance between January 2013 to December 2016. The sampling design assigned a sample weight to each beneficiary. Weighted descriptive analyses, Poisson regression and generalized linear model were used to analyze the medical service utilization, costs and their associations with patient characteristics.

**Results:** In urban China, the annual prevalence of stroke was 730.43 (95% CI = 730.10-730.76) cases per 100 000 people, and nearly 2% of total health expenditures of urban residents was spent on stroke-related medical costs. Weighted average annual total medical cost of stroke was RMB10 637 [95% CI = 10 435-10 840] (US$1682, 95% CI = 1650-1714), with annual out-of-pocket (OOP) cost of RMB3093 [95% CI = 3026-3161] (US$489, 95% CI = 478-500). The average yearly number of stroke-related outpatient visit was 1.67 [SD = 3.39] and inpatient admission was 0.79 [SD = 0.83], with an average cost of RMB440 [SD = 739] (US$70, SD = 117) for outpatients and RMB12 702 [SD = 21 424] (US$2008, SD = 3387) for inpatients. Inpatient costs accounted for 94% (RMB10 034 or US$ 1586) of medical costs, and tertiary hospitals were the main provider of stroke care. Stroke-related medical care utilization and direct costs were associated with gender, age, pathological stroke types and insurance status. Medication costs contributed to 50.6% (RMB5382 or US$ 851) of the average stroke-related medical costs.

**Conclusion:** China’s health system bares a large economic burden from stroke. Specific policies are needed to strengthen the capacity of secondary hospitals, alter the structure of medical resource allocation, and target specific sections of the stroke population.

## Background

**Key Messages**

**Implications for policy makers**

- This study depicts a full view of direct medical cost, and medical resource utilizations of stroke in urban China, which will be highly useful for the assessment of the cost-effectiveness of specific interventions and the allocation of resources related to stroke prevention, diagnosis and treatment.
- In urban China, the annual prevalence of stroke was 730.43 cases per 100 000 people, with nearly 2% of total health expenditures of urban residents spent on stroke, and the average annual direct medical cost per stroke was RMB10 637 (US$1682), with out-of-pocket (OOP) costs of RMB3093 (US$489) or 29.04% of total stroke costs.
- Inpatient costs were the major component (95%) of stroke-related medical costs, and medication costs were the major contributor (50.6%) to average stroke-related medical costs.

**Implications for the public**

This research provides recommendations for strengthening the capacity of secondary hospitals, altering the structure of medical resource allocation, and targeting specific sections of the stroke population, which will improve population health outcomes, and reduce the financial burden on patients with stroke.

Stroke is the second most common cause of death globally, imposing a heavy financial burden on patients and a country’s health system.^1^ Cost of illness studies reported that the healthcare expenditures spent on strokes is roughly 2%-4% of total health expenditures worldwide and accounted for more than 4% of total health expenditures in developed countries.^2,3^ The total annual direct costs of stroke have been variously estimated at £4 billion in the United Kingdom,^4^ €5 billion in France,^5^ and US$21 billion in the United States,^2,6^ with the estimated average cost of stroke US$19 018 (median US$14 571), ranging from US$468 to US$146 149.^7^

China bears the largest cost burden of stroke in the world. In China, stroke is the leading cause of death, with an estimated 1.1 million stroke-related deaths in 2013 or about 15% of the global total of 7 million stoke deaths.^8^ Epidemiological surveys have highlighted a marked increase in the prevalence and incidence of stroke in the past decade,^8-10^ with an estimated 11 million prevalent cases and 2.4 million new cases in 2013.^9^ With population ageing and an ongoing high prevalence of risk factors, the burden of stroke is expected to further increase in China.

Surprisingly, a very limited number of studies have analyzed the treatment cost of stroke in China. A study conducted in China estimated the direct medical cost for ischemic stroke was RMB23.7 (US$3.4) billion and for intracerebral hemorrhage RMB13.7 (US$2.0) billion in 2003.^11^ The average medical cost of per stroke inpatient admission ranged from RMB6773 ($US983) in Sichuan^12^ to RMB30 550 (US$4434) in Beijing.^13^ These estimates of the treatment costs of stroke have several limitations. First, most treatment cost studies were regional with small sample sizes, which constrain drawing conclusions from national perspective. Second, the first nationwide study was conducted a decade ago and based on admission of only government-funded general hospitals.^11^

With the rapid increase of aging population, increased health insurance coverage and newer therapeutic modalities, an updated estimate of the treatment costs of stroke in China is urgently needed. In the past decade, total health expenditure in China grew at a rate of 15%, which was almost twice the growth rate of gross domestic product (GDP). Cost containment is a key aspect of China’s health system reforms. It is estimated that cardiovascular and cerebrovascular diseases accounted for 20.7% of the increase in health expenditures.^14^ A comprehensive stroke analysis, including the demographic characteristics of stroke patients and their patterns of inpatient and outpatient services use and costs, will better define the stroke patient burden on China’s healthcare system, help analyze the cost-effectiveness of specific interventions, and guide the allocation of resources related for stroke prevention, diagnosis, and treatment.

 Using China’s Urban Employee’s Basic Medical Insurance (UEBMI) and Urban Resident’s Basic Medical Insurance (URBMI) claims data between January 2013 to December 2016, this study aims to estimate the annual prevalence and direct medical cost of stroke in urban China. Also, this study assesses stroke-related medical care utilization (outpatient visits and inpatient admissions) and direct stroke medical costs, and their association with stroke patient sociodemographic characteristics and stroke pathological type. In addition, the study investigates the structure of stroke medical resource use.

## Methods

###  Data Source 

The data we obtained comprise a 5% random sample of UEBMI and URBMI beneficiaries’ insurance claims, which used stratified random sample design and were conducted by the China Health Insurance Research Association.^15^ UEBMI and URBMI are the two main health insurance schemes administrated by China government. During the 2013-2016 period, the two schemes covered more than 95% of the residents in urban China, accounting for roughly 750 million, or 53%, of the total Chinese population.^16^ The data contain stroke beneficiaries’ demographic information, diagnoses and cost of hospital admissions and outpatient visits. The sampling design assigned a sample weight to each beneficiary, which is equal to the reciprocal of the selection probability to correct for systematic differences in probability sampling. The use of these weights allowed us to estimate national medical care utilization and costs in stroke patients.

 The total population count of insured was obtained from China labor statistical yearbook. The age and gender structure were based on the annual national sample survey of population changes.

###  Samples 

 Based on the International Classification of Diseases, 10th version (ICD-10) codes (I60, I61, I62, I63, and I64 as the primary diagnosis), this study identified 371 891 stroke patients with 633 388 stroke-related medical records between 2013 and 2016. Subtypes of stroke were classified as ischemic stroke (ICD-10: I63), hemorrhagic stroke (ICD-10: I60–I62) and undetermined stroke (ICD-10: I64).

###  Measures 

 Medical care utilization included the number of annual outpatient visits (including pharmacy, primary care, secondary and tertiary hospitals) and the number of annual inpatient admissions (including primary care facilities and secondary and tertiary hospitals). Direct medical costs were measured by the average cost per visit and average out-of-pocket (OOP) cost per visit. All the cost, including spending on medication, medical services, diagnostic tests/medical consumables, were measured in RMB and converted to US$ at the exchange rate RMB/US$ = 0.158. OOP spending means the direct cash payment that may not be reimbursed by health insurance, including deductibles, coinsurance, copay expenses and costs of drugs and tests not included in the insurance list.

 Control variables included stroke type (ischemic, hemorrhagic and undetermined), age groups (younger than 20 years, 20-29 years, 30-39 years, 40-49 years, 50-59 years, 60-69 years, 70-79 years, and 80 years or older), gender (male and female), insurance type (UEBMI and URBMI) and year (2013, 2014, 2015, and 2016).

###  Statistical Analysis

 To estimate the prevalence of stroke, this study counted the total number of beneficiaries in the database who met the definition of stroke and subtypes strokes, and their basic demographic characteristics as the numerator. The total population count of insured was the denominator. Weighted descriptive analyses were used to analyze the sample characteristics, medical service utilization and costs. Associations between medical service utilization and stroke type and sociodemographic characteristics were evaluated by Poisson regressions. The generalized linear model with a gamma distribution and a log link was used to assess the association of average total costs and OOP cost with sociodemographic characteristics and stroke type. Since the primary interest of this study was to assess stroke-related medical service utilization and direct stroke medical costs, the coefficients were transformed back to the original scale. Therefore, to calculate annual visits or cost for any patient, the addition factors for each of the characteristics for that patient were added onto a baseline estimate (female, age less than 20, URBMI, ischemic stroke in 2013).

Unless otherwise specified, all descriptive statistics and generalized linear model estimates in the text and tables are weighted to have national estimates based on the sample weight provided by the China Health Insurance Research Association. A *P* value of less than 0.05 was considered statistically significant. The software Stata version 15 for Windows (StataCorp, College Station, TX, USA) was used for the statistical analysis.

## Results

###  Sample Characteristics

Table 1 displays the sample characteristics, prevalence and direct medical cost with stroke by pathological type, gender, age and insurance type. There were 371 891 stroke patients in the sample, and the weighted number were 18 826 762. Among the weighted sample, ischemic was the main subtype of stroke, accounting for 76.15% of all strokes. Among all stroke patients, 54.89% was male, 73.48% was aged 60 years or older, and 67.91% was covered by UEMBI.

**Table 1:** Characteristics of Sample, Prevalence and Direct Medical Cost of Stroke, 2013 to 2016

	**Sample, Thousands (%)**	**Weighted Sample, Thousands (%)**	**Annual Prevalence, 1/100 000 (95% CI)**	**Annual Direct Medical Cost Per Person, RMB (95% CI)**	**Annual OOP Per Person, RMB (95% CI)**	**Total Direct Medical Cost, Billion RMB (95% CI)**
Pathological type						
Ischemic	246.64 (66.32)	14 336.83 (76.15)	556.23 (555.95, 556.52)	9682 (9511, 9852)	2716 (2661, 2770)	138.8 (136.3, 141.2)
Hemorrhagic	104.74 (28.16)	2724.50 (14.47)	105.70 (105.58, 105.83)	20 007 (18 957, 21 058)	6459 (6075, 6843)	54.8 (52.0, 57.7)
Undetermined	20.51 (5.52)	1765.43 (9.38)	68.49 (68.39, 68.60)	3854 (3375, 4333)	936 (836, 1035)	6.8 (6.0, 7.7)
Gender						
Female	164.79 (44.31)	8492.15 (45.11)	673.41 (672.96, 673.86)	9293 (9034, 9552)	2889 (2798, 2980)	79.0 (76.8, 81.2)
Male	207.10 (55.69)	10 334.61 (54.89)	785.06 (784.58, 785.54)	11 742 (11 442, 12 042)	3261 (3164, 3359)	121.5 (118.4, 124.6)
Age group						
0-19	0.54 (0.15)	45.56 (0.24)	9.83 (9.74, 9.92)	8762 (6009, 11 514)	4394 (2808, 5979)	0.4 (0.3, 0.5)
20-29	7.00 (1.88)	124.04 (0.66)	24.48 (24.35, 24.62)	9808 (6256, 13 360)	3054 (1945, 4163)	1.2 (0.8, 1.7)
30-39	15.75 (4.24)	342.42 (1.82)	75.26 (75.01, 75.51)	9345 (7938, 10 752)	2891 (2419, 3363)	3.2 (2.7, 3.7)
40-49	35.51 (9.55)	1262.80 (6.71)	272.11 (271.63, 272.58)	11 077 (9766, 12 388)	4003 (3462, 4544)	14.0 (12.4, 15.7)
50-59	72.57 (19.51)	3299.43 (17.53)	1006.32 (1005.24, 1007.41)	10 169 (9787, 10 550)	3333 (3190, 3475)	33.6 (32.3, 34.9)
60-69	98.04 (26.36)	5321.99 (28.27)	2505.90 (2503.80, 2508.00)	10 067 (9715, 10 418)	3059 (2933, 3185)	53.6 (51.7, 55.5)
70-79	92.47 (24.86)	5361.18 (28.48)	5185.50 (5181.23, 5189.78)	10 567 (10 183, 10 950)	2846 (2740, 2952)	56.7 (54.6, 58.7)
80+	50.02 (13.45)	3069.34 (16.30)	6849.99 (6842.59, 6857.39)	12 280 (11 802, 12 757)	2957 (2840, 3075)	37.7 (36.2, 39.2)
Insurance						
URBMI	80.06 (21.53)	6041.82 (32.09)	420.66 (420.32, 420.99)	10 325 (9953, 10 697)	4356 (4197, 4515)	62.4 (60.1, 64.6)
UEBMI	291.83 (78.47)	12 784.94 (67.91)	1119.88 (1119.27, 1120.49)	10 785 (10 542, 11 027)	2498 (2438, 2557)	138.1 (135.0, 141.2)
Total	371.89 (100.00)	18 826.76 (100.00)	730.43 (730.10, 730.76)	10 637 (10 435, 10 840)	3093 (3026, 3161)	200.5 (196.6, 204.3)
Total health expenditure of urban residents (2013 to 2016), Billion RMB						11697.7
Total direct medical cost as percent of total health expenditure of urban residents, %						1.7
Average GDP per capita between 2013 to 2016, RMB						48599
Average annual direct medical cost as percent of GDP per capita, %						21.9
Average OOP cost as percent of disposable income among urban residents, %						9.9

Abbreviations: OOP, out-of-pocket; UEBMI, Urban Employee’s Basic Medical Insurance; URBMI, Urban Resident’s Basic Medical Insurance; GDP, gross domestic product.

###  Annual Prevalence and Direct Medical Cost

Annual prevalence of stroke was 730.43 (95% CI = 730.10-730.76) cases per 100 000 people during 2013 to 2016. The most prevalent type of stroke was ischemic which was 556.23 (95% CI = 555.95-556.52) cases per 100 000 people. The annual prevalence was higher among men (785.06/100 000, 95% CI = 784.58/100 000-785.54/100 000) than women (673.41/100 000, 95% CI = 672.96/100 000-673.86/100 000). Urban Chinese aged 80 or older had the highest prevalence (6849.99/100 000, 95% CI = 6842.59/100 000-6857.39/100 000). Prevalence of stroke (with 95% CI) in China in 2013 to 2016 by age and gender are displayed in Supplementary file 1 (Figure S1).

The weighted average annual direct medical cost of stroke was RMB10 637 [95% CI = 10 435-10 840] (US$1682, 95% CI = 1650-1714), with 29.04%, or RMB3093 [95% CI = 3026 -3161] (US$489, 95% CI = 478-500), of total costs OOP expenses. Patients with hemorrhagic stroke had the highest annual direct medical cost of RMB20 007 [95% CI = 18 957-21 058] (US$3163, 95% CI = 2997-3329), with the highest OOP percentage of 32.28%. Male patients have higher annual direct medical cost than female patients (RMB11 742 VS. 9293, *P*< .001). Cost in US$ and Euro are presented in Supplementary file 1 (Table S1).

 The estimated total direct medical cost for stroke in urban China was RMB200.5 [95% CI = 196.6-204.3] (US$31.8, 95% CI = 31.1-32.3) billion during 2013 to 2016 (roughly RMB50.1/ US$8.0 billion per year). The cost of cerebral infarction (ICD-10 code: I63) was the highest (RMB138.8/ US$21.9 billion). The cost for male patients was 1.5 times that for female patients. Cost in patients aged 60 years and older accounted for 74% of total direct medical cost of stroke.

 To evaluate the impact of stroke-related direct medical cost on the health system, economy and personal consumption, the proportion of health expenditure spent on stroke in total health expenditure of urban residents was 1.7%; average annual direct medical cost per patient accounted for 21.9% of GDP per capita, and the average annual OOP spending accounted for 9.9% of total disposable income among urban residents during 2013 to 2016.

###  Annual Utilization of Medical Care and Associated Costs

An overview of medical utilization, outpatient visits and inpatient admissions, and their associated costs, are presented in Table 2. The annual number of outpatient visits per stroke patient was 1.67, incurring, on average, costs of RMB440 [standard deviation, SD = 739] (US$ 70, SD = 117) per visit, or the annual cost of outpatient treatment of RMB559 (US$88). Inpatients admissions were 0.79 [SD = 0.83] per stroke patient, incurring an average cost of RMB 12 702 [SD = 21 424] (US$2008, SD = 3387) per inpatient, or an average annual cost of inpatient treatment of RMB10 034 (US$1586). Patients with undetermined stroke had the highest utilization of outpatient services, while patients with ischemic and hemorrhagic stroke had the highest rate of inpatient service utilization. As shown in Table 2, patients used more inpatient services in secondary and tertiary hospitals than in primary care facilities. Patients with hemorrhagic stroke had the highest average cost per inpatient admission, RMB26 847 [SD = 44 179] (US$4244, SD = 6984), followed by patients with undetermined stroke RMB11 412 [SD = 17 253] (US$1804, SD = 2727) and ischemic stroke RMB10 472 [SD = 13 685] (US$1655, SD = 2163). Tertiary hospitals had higher average cost than primary clinics and secondary hospitals. OOP cost displayed a similar pattern to total costs by stoke type and care facility. Cost in US$ and the Euro are presented in Supplementary file 1 (Table S2).

**Table 2:** Summary of Annual Utilization of Medical Care and Associated Costs (n = 18 826 762)

	**Average Annual Visit Number**				**Average Cost Per Visit, RMB**				**Average OOP Cost Per Visit, RMB**			
		**Ischemic**	**Hemorrhagic**	**Undetermined**	**Total**	**Ischemic**	**Hemorrhagic**	**Undetermined**	**Total**	**Ischemic**	**Hemorrhagic**	**Undetermined**	**Total**
Outpatient	1.58 (3.50)	1.72 (2.96)	2.15 (3.22)	1.67 (3.39)	456 (720)	427 (1050)	386 (312)	440 (739)	193 (292)	130 (527)	103 (173)	169 (330)
Pharmacies	0.19 (1.47)	0.72 (1.53)	0.01 (0.39)	0.25 (1.41)	509 (287)	312 (405)	356 (440)	420 (359)	379 (224)	41 (109)	61 (127)	226 (248)
Primary care	0.44 (1.75)	0.28 (1.63)	0.47 (1.78)	0.42 (1.73)	331 (447)	433 (448)	316 (315)	339 (433)	128 (207)	131 (165)	69 (141)	120 (196)
Secondary hospital	0.59 (2.00)	0.32 (1.49)	0.95 (2.18)	0.59 (1.96)	441 (527)	447 (568)	351 (222)	421 (483)	177 (243)	158 (166)	90 (113)	154 (212)
Tertiary hospital	0.36 (1.63)	0.40 (1.81)	0.71 (2.19)	0.41 (1.74)	542 (1010)	656 (2206)	449 (355)	533 (1084)	192 (360)	293 (1163)	131 (219)	188 (468)
Inpatient	0.87 (0.81)	0.76 (0.95)	0.25 (0.61)	0.79 (0.83)	10 472 (13 685)	26 847 (44 179)	11 412 (17 253)	12702 (21424)	2819 (4966)	8784 (21 279)	3027 (5105)	3629 (9308)
Primary care	0.15 (0.45)	0.08 (0.34)	0.04 (0.23)	0.13 (0.42)	5750 (8938)	11 842 (23 840)	4454 (6486)	6262 (11 199)	1229 (2479)	2952 (8995)	950 (1424)	1376 (3595)
Secondary hospital	0.36 (0.64)	0.29 (0.69)	0.10 (0.40)	0.33 (0.63)	8024 (8413)	17 635 (31 888)	10 765 (15 870)	9250 (14 134)	2134 (3109)	5405 (15 384)	2655 (3671)	2544 (6216)
Tertiary hospital	0.36 (0.63)	0.39 (0.72)	0.11 (0.39)	0.34 (0.63)	14 836 (17 713)	36 379 (51 921)	14 727 (20 217)	18 374 (27 765)	4148 (6630)	12 345 (25 550)	4175 (6604)	5495 (12 379)

Abbreviation: OOP, out-of-pocket. Standard deviation in parentheses.

###  Predictors of Medical Service Utilization and Costs 

Medical service utilization (outpatient visits or inpatient admissions) and costs associated with each utilization by sociodemographic characteristics and pathological type are presented in Table 3. The detailed results of the multivariate analyses and the cost values in US$ and Euro are presented in Supplementary file 1 (Table S3 and Table S4). The baseline represents utilization and medical costs for an under 20 year old female with URBMI and ischemic stroke in 2013. When a patient displays any of the characteristics listed in Table 3, the annual utilization and medical costs are estimated by the sum of the baseline and the addition costs corresponding to each of the patient’s characteristics. For example, the annual number of outpatient visits for an under 20 year old female with URBMI and ischemic stroke in 2013 was 0.521, and for a male was 0.669 (0.521+0.148).

**Table 3:** Incremental Utilization and Medical Cost Associated With Sociodemographic Characteristics, Pathological Type and Year (n = 18 826 762)

	**Outpatient**			**Inpatient**		
	**The Average Number of Annual Visits**	**Average Cost Per Visit (RMB)**	**Average OOP Cost Per Visit (RMB)**	**The Average Number of Annual Admission**	**Average Cost Per Admission (RMB)**	**Average OOP Cost Per Admission (RMB)**
**Baseline**	0.521*** (0.515, 0.527)	208.8*** (205.0, 212.7)	100.8*** (97.9, 103.8)	0.618*** (0.610, 0.625)	6304.5*** (6202.4, 6406.6)	3215.3*** (3142.5, 3288.0)
**Increment**						
Pathological type						
Hemorrhagic	-0.005*** (-0.007, -0.003)	-26.2*** (-27.0, -25.4)	-61.8*** (-62.2, -61.4)	-0.053*** (-0.054, -0.051)	16873.6*** (16 821.7, 16 925.5)	5626.9*** (5604.6, 5649.2)
Undetermined	0.642*** (0.639, 0.644)	-71.2*** (-71.9, -70.4)	-83.3*** (-83.7, -83.0)	-0.605*** (-0.606, -0.604)	978.3*** (923.0, 1033.6)	224.1*** (203.4, 244.8)
Gender						
Male	0.148*** (0.147, 0.149)	-0.8** (-1.4, -0.3)	10.5*** (10.2, 10.8)	0.145*** (0.145, 0.146)	431.0*** (413.1, 448.9)	30.0*** (22.8, 37.2)
Age group						
20-29	-0.339*** (-0.358, -0.321)	-31.1*** (-36.8, -25.3)	-16.1*** (-20.6, -11.6)	-0.187*** (-0.194, -0.181)	13558.0*** (13 197.2, 13 918.7)	2278.2*** (2141.2, 2415.2)
30-39	-0.376*** (-0.394, -0.358)	-22.6*** (-28.2, -17.1)	-15.5*** (-19.9, -11.2)	0.025*** (0.018, 0.031)	3745.5*** (3564.3, 3926.7)	793.5*** (706.0, 881.0)
40-49	-0.117*** (-0.135, -0.099)	82.4*** (77.0, 87.9)	14.3*** (10.0, 18.6)	0.198*** (0.192, 0.204)	2528.5*** (2361.9, 2695.1)	642.6*** (560.7, 724.4)
50-59	0.244*** (0.227, 0.262)	150.7*** (145.3, 156.1)	28.3*** (24.1, 32.6)	0.255*** (0.249, 0.261)	1657.5*** (1493.3, 1821.7)	203.8*** (123.0, 284.5)
60-69	0.280*** (0.263, 0.298)	161.4*** (156.0, 166.8)	27.0*** (22.7, 31.2)	0.305*** (0.299, 0.311)	1826.2*** (1662.3, 1990.1)	-48.8 (-129.4, 31.7)
70-79	0.219*** (0.202, 0.237)	167.4*** (162.0, 172.8)	22.7*** (18.4, 26.9)	0.350*** (0.344, 0.356)	2513.6*** (2349.6, 2677.6)	-109.6** (-190.1, -29.0)
80+	0.061*** (0.044, 0.079)	154.3*** (148.9, 159.8)	12.7*** (8.5, 16.9)	0.381*** (0.375, 0.387)	4651.9*** (4486.7, 4817.1)	-24.3 (-105.0, 56.5)
Insurance						
UEBMI	1.182*** (1.181, 1.183)	190.4*** (189.8, 190.9)	25.8*** (25.4, 26.3)	-0.235*** (-0.236, -0.234)	3531.5*** (3514.2, 3548.8)	-1301.5*** (-1309.5, -1293.5)
Year						
2014	-0.047*** (-0.049, -0.046)	-75.7*** (-76.7, -74.6)	48.7*** (48.2, 49.2)	-0.110*** (-0.111, -0.109)	361.9*** (336.2, 387.5)	220.2*** (209.7, 230.8)
2015	0.627*** (0.625, 0.629)	-42.2*** (-43.2, -41.2)	49.3*** (48.9, 49.8)	-0.083*** (-0.084, -0.082)	1073.8*** (1049.4, 1098.3)	155.3*** (145.6, 165.0)
2016	0.730*** (0.728, 0.732)	-54.8*** (-55.7, -53.8)	60.5*** (60.1, 61.0)	-0.041*** (-0.043, -0.040)	1548.3*** (1524.2, 1572.4)	367.8*** (358.2, 377.5)

Abbreviations: OOP, out-of-pocket; UEBMI, Urban Employee’s Basic Medical Insurance; URBMI, Urban Resident’s Basic Medical Insurance. The baseline represents the utilization and cost for an under 20-year-old female with Urban Residents’ Basic Medical Insurance and ischemic stroke in 2013. 95% CI in parentheses; *** *P* < .001, ** *P* < .010.

 Although the number of annual visits of outpatient patients with undetermined stroke was 0.642 (95% CI = 0.639-0.644) times more than the baseline patients with ischemic stroke, the average cost per visit was RMB71.2 [95% CI = 70.4-71.9] (US$11.2, 95% CI = 11.1-11.4) less than that of ischemic stroke patients. The number of annual visits of inpatient hemorrhagic stroke was 0.053 (95% CI = 0.051-0.054), and for undetermined stroke 0.605 (95% CI = 0.604-0.606), times less than that of baseline patients with ischemic stroke, with an average cost per admission of RMB16 873.6 [95% CI =16 821.7-16 925.5] (US$2667.4, 95% CI = 2659.2-2675.6) for hemorrhagic and RMB978.3 [95% CI = 923.0-1033.6] (US$154.7, 95% CI = 145.9-163.4) for undetermined stroke greater than baseline patients with ischemic stroke.

 Men utilized more inpatient services with higher average cost per admission than women. Compared to younger patients, patients aged 50 or above used more outpatient and inpatient services, with a high average cost per visit and admission. The total inpatient cost per admission was higher in patients covered by UEBMI than patients covered by URBMI, while the OOP cost for UEBMI patients was lower than URBMI patients. Compared with 2013, the average costs of outpatient visits decreased 2014-2016, while average costs rose for inpatients.

###  Structure of Medical Resource Use

The distribution of various medical costs by pathological stroke type are presented in Figure. The highest cost was medication, which accounted for 50.6% (RMB5382 or US$ 851) of total medical costs, while diagnostic tests and medical consumable cost was the smallest (9.5%, RMB1011 or US$160) proportion of medical costs. Ischemic and undetermined stroke patients had similar patterns of resource use (*P*= .735), with both ischemic (*P* <.001) and undetermined (*P* <.001) stroke patients having higher medication costs than hemorrhagic patients.

**Figure:**
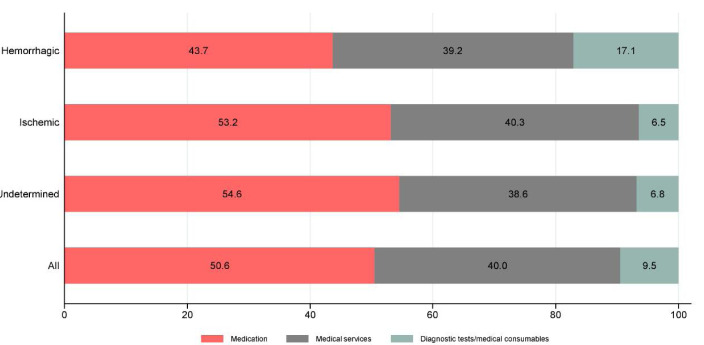
Cost Subtypes Distribution of Patients With Stroke by Pathological Type (Percentages).

## Discussion

 Based on health insurance claim data, this study provides a comprehensive estimate of stroke-related medical care utilization and direct medical costs in urban China. In the sample of nearly 372 thousand patients with stroke, this study found that the annual prevalence of stroke was 730.43 cases per 100 000 people, and nearly 2% of total health expenditures of urban residents was spent on stroke in urban China. The average annual direct medical cost of stroke was RMB10 637 (US$1682), with OOP costs of RMB3093 (US$489) or 29.04% of total stroke costs. The average number of stroke-related outpatient visits was 1.27 and inpatient admissions was 0.79, with an average cost of RMB440 (US$70) per outpatient and RMB12 702 (US$2008) per hospital admission. Inpatient costs were the major component (94%) of stroke-related medical costs. Compared to ischemic stroke patients, patients with hemorrhagic and undetermined stroke used fewer inpatient services, but with a high average cost per admission. Men and elderly utilized more inpatient services with higher average cost per admission than women and younger. The total inpatient cost per admission was higher for UEBMI than URBMI patients, while OOP costs were higher for URBMI than UEBMI patients. Medication costs were the major contributor to average stroke-related medical costs.

The prevalence of stroke in this study was less than that in some other epidemiological studies.^8,9^ The claim data did not count stroke patients who did not go to healthcare facilities or make an insurance claim, but this was not expected to be large since most stroke patients attended hospital services.^17^ In addition, the medical information system in China is undergoing upgrades, especially for outpatient services, so there may be some inaccuracy or incompleteness in patients’ medical information.

In this study, roughly 2% of total health expenditures of urban residents was spent on stroke, which was slightly lower than that estimated in developed countries.^2^ There are two potential reasons. First, this study only included the direct medical cost, which did not include the cost of long-term care and family care. Second, this study did not estimate the cost of stroke in rural areas. In China, total health expenditure of urban residents accounts for 77% of national total health expenditure while the population in urban area only accounted for 56% of total population in 2015.^18^

The annual cost per stroke patient in our study is lower than those reported in the Organisation for Economic Co-operation and Development (OECD) countries^7,19^ and some other Asian countries, such as Singapore.^20^ Potential reasons for the difference were the type of costs included in the non-Chinese studies, such as non-medical cost and indirect cost, the number of pathological types and differences in the economic level of development. The estimates in this study were also lower than another study in China, which found the average cost of stoke was RMB30438 (US$4812).^21^ However, the previous study only estimated the first year (acute) stroke medical costs,^19,22^ which means the first year average cost was much higher than the longer-term average annual costs estimated in this study. In urban China, the OOP expense was roughly 10% of the total income among urban residents, which is less than that in other Asian countries, such as India.^23^ This can be largely explained by the near universal population coverage of social health insurance and the high proportion of insurance reimbursement.^16^ Consistent with previous studies, patients with hemorrhagic stroke had higher annual medical cost.^20^ This is due to hemorrhagic stroke patients frequently needing neurosurgery and intensive monitoring with high levels of subsequent morbidity.^19,24^

In this study, inpatient costs were the major component of medical expenses, which is consistent with previous studies.^25,26^ Given that many complications frequently accompany stroke, it is unsurprising that inpatient care dominates the direct medical costs of stoke.^20^ Stoke hospitalizations were mainly in secondary and tertiary hospitals, and both total cost and OOP costs of inpatient services in tertiary hospitals are roughly twice that in secondary hospitals, partly due to significant differences in technology and equipment between tertiary and secondary hospitals.^27^ This fact underlines the need to strengthen the capacity of secondary hospitals and increasing the proportion of secondary hospitals that have certified stroke centers. In terms of outpatient costs, the utilization and average medical costs showed little difference between facilities.

Consistent with previous studies, this study found significant differences in medical service utilization and costs by stroke types, gender and age.^23,28^ Patients with hemorrhagic stroke made fewer hospital visits per year, but had a higher cost per admission than ischemic stroke patients. Men used more medical services with higher cost per visit than women. Elder patients had higher utilization of hospitalization with greater cost per visit than the non-elderly. Matching these findings with clinical and epidemiological data are useful in identifying targets for stroke prevention and developing specific cost-effective stroke programs. Given higher stroke prevalence and incidence in males than females, combined with higher medical expenditures for male stroke patients, preventative and educational programs targeted to men are cost-effective health interventions. Important risk factors of stroke, such as smoking, diet, lifestyle and alcohol use, are different between men and women.^8,9^ So more intensive action in public education, advertising regulations and tax policies on tobacco, diets and alcohol use, and direct governmental interventions to control smoking and alcohol use, are important and cost-effective stroke-mitigation strategies.

Disparities between the two insurance types were observed. The total inpatient cost per admission was higher for patients covered by UEBMI than URBMI patients, while the OOP cost was lower for UEBMI than URBMI patients. This is mainly due to the disparities in insurance cover, benefits and reimbursement rates. Since UEBMI had higher premiums, it covered more services and medications than URBMI, and also had a higher reimbursement rate (UEBMI 68% versus URBMI 48%).^29^ The potential reason for UEBMI-URBMI differences was the target population. The UEBMI targeted urban employees while URBMI targeted urban children, students, unemployed, and the disabled.^29^ Since elderly and disabled people, with a high prevalence of comorbidity and complications, were covered by URBMI, the inpatient admission rate was high relative to the UEBMI group. In addition, the higher number of medical services covered and the higher reimbursement rate in UEBMI and URBMI lead to the increase of the average costs of inpatient during 2013 to 2016.

In this study, more than half of the direct medical cost was attributed to medication, which is consistent with other studies in China.^12,13^ However, medication costs accounted for less than 10% of stroke costs in developed countries, such as German^22^ and Korea.^19,30^ This might be explained from both the demand and supply side. From the supply side, the drug mark-up policy before 2015 and bonus system which rewarded physicians based on the monetary values of drugs they prescribed, encouraged physicians to over-prescribe medicines.^31^ Beginning 2009, the zero-markup drug policy ended the previously permitted 15% profit margin for drug sales, which has been progressively piloted across China at community health centers (2009), county public hospitals (2012) and urban public hospitals (2015). Although previous research has found a significant drop in post-2009 medication costs, for stoke-related drugs the proportion in total stoke costs declined only slightly. This may be related to the type of drug treatment required for stoke patients or it may that more effective incentives should be developed to change physicians’ drug prescription habits. From the demand side, Chinese patients usually pay much attention to their medication therapy when they get ill,^12^ demanding drug prescriptions. Also, the wide use of traditional Chinese medicine encourages the demand and support of medications in China in contrast to western and other Asian countries.^32^

 This study has several policy implications. First, the China’s health system bares a huge economic cost stroke burden. This requires specific policies to contain the escalating stroke medical costs. Second, the government should design policies to strengthen the capacity of secondary hospitals to treat stroke cases and increase the proportion of secondary hospitals that have certified stroke centers. The increased quality in secondary hospitals will increase its utilization to deal with a variety of stroke patients, decrease the mortality-to-incidence ratio and the contain medical stroke costs. Third, specific and cost-effective interventions could be developed based on the detailed cost estimations in this study, especially related to drug prescriptions. In addition, more effective incentives should be developed to change physicians’ drug prescription habits and patients’ thoughts on drug use. Finally, this study recommends targeted education campaigns and government interventions to address alcohol, smoking and diet risk-factors as stroke-mitigation strategies, which vary between men and women.

There are some limitations in this study. First, the data do not allow us to separate the cost of the first-year cost from that for the following years. Second, the estimates in this study do not take into account of the severity of stroke due to a lack of related clinical information. Third, since the data were claim data, patients who did not go to healthcare facilities were not included in the sample. Fourth, the inaccuracy and incompleteness of patients’ medical information may have affected the estimates. However, 97.07% of hospitals used ICD-10 codes, which was determined by a qualified doctor, providing confidence in the reliability of stroke coding.^33^ Despite these limitations, the strengths of UEBMI and URBMI claims data was the ability to estimate stroke-related medical care utilization and the direct stroke medical costs and, as well as to provide a comprehensive cost analysis for stroke patients in urban China.

## Conclusion

 China’s health system bares a large economic stroke burden, with tertiary hospitals the main provider of medical services. Stroke type, gender and age were significant determinants of the total medical cost burden of stroke treatment. This study observed an unreasonable cost structure, with medication costs significantly higher than OECD and other Asian countries. With an aging population, the prevalence of stroke in China will continue to grow, resulting in an escalating burden on health budgets. Hence, it is important to understand what drives the rapid growth of stroke medical costs in order to further underscore the need for effective preventive therapies and timely critical care programs, such as strengthening the capacity of secondary hospitals to treat strokes, increase the proportion of secondary hospitals that have certified stroke centers, and alter the structure of medical resource allocation to contain stroke-related medical costs and to enhance the quality of life of China’s aging society.

## Ethical issues

 Since the claims data were an anonymized database and had no influence on patient care, the Ethics Committee of Beijing University of Chinese medicine deemed this study as exempt from ethical approval (No. 2019BZHYLL0201).

## Competing interests

 Authors declare that they have no competing interests.

## Authors’ contributions

 DZ, XS, and PH participated in the study concept, design. DZ and PH performed the statistical analysis and drafted the first version of the manuscript. SN, SC, RD, LH, and YM performed critical revision of article for important intellectual content. All authors contributed to the data interpretation.

## Funding

 This paper was supported by Peking University’s Start-up Fund (No. BMU2018YJ004).

## Authors’ affiliations

^1^China Center for Health Development Studies, Peking University, Beijing, China. ^2^School of Management, Beijing University of Chinese Medicine, Beijing, China. ^3^National Institute of Chinese Medicine Development and Strategy, Beijing University of Chinese Medicine, Beijing, China. ^4^School of Economics and School of Management, Tianjin Normal University, Tianjin, China. ^5^Australian National Institute of Management and Commerce, Sydney, NSW, Australia. ^6^Research Institute for International Strategies, Guangdong University of Foreign Studies, Guangzhou, China. ^7^Newcastle Business School, University of Newcastle, Newcastle, NSW, Australia. ^8^School of Public Health, Peking University, Beijing, China. ^9^Institute of Population Research, Peking University, Beijing, China. ^10^China Health Insurance Research Association, Beijing, China.

## Supplementary files


Supplementary file 1 contains Figure S1 and Tables S1-S4.
