# Supplementary material for: Medical Service Utilization and Direct Medical Cost of Stroke in Urban China
Source: Int J Health Policy Manag. 2020 Jul 20;11(3):277–86. doi: 10.34172/ijhpm.2020.111 (PMC9278468; doi:10.34172/ijhpm.2020.111)
Supplement: Supplementary file 1 — contains Figure S1 and Tables S1-S4. [file ijhpm-11-277-s001.pdf]

Supplementary file 1

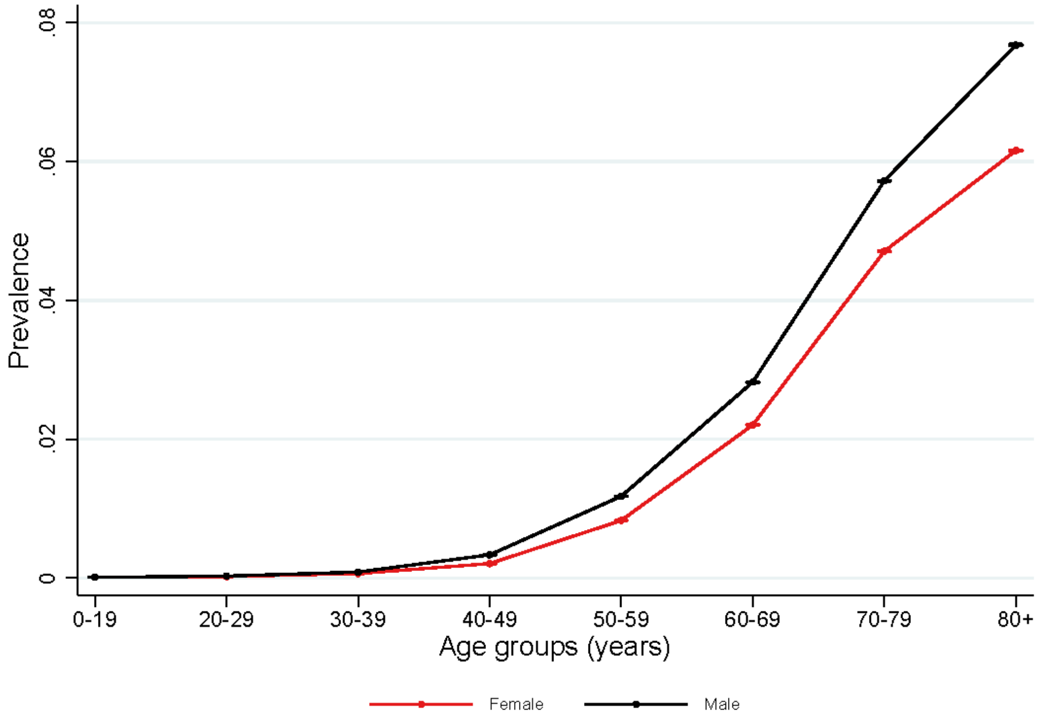

Figure S1. Prevalence of stroke (with 95% CI) in urban China in 2013 to 2016 by age and sex.

Table S1. Direct medical expenditure of stroke in USD and EUR respectively

	Annual direct medical cost per person, USD	Annual OOP per person, USD	Total direct medical cost, Billion USD	Annual direct medical cost per person, EUR	Annual OOP per person, EUR	Total direct medical cost, Billion EUR
Pathological type						
Ischemic	1530.5(1503.5,1557.5)	429.3(420.7,437.9)	21.9(21.6,22.3)	1263.1(1240.8,1285.4)	354.3(347.2,361.4)	18.1(17.8,18.4)
Hemorrhagic	3162.8(2996.7,3328.9)	1021.1(960.4,1081.8)	8.7(8.2,9.1)	2610.3(2473.3,2747.4)	842.7(792.7,892.8)	7.2(6.8,7.5)
Undetermined	609.3(533.6,685.0)	147.9(132.2,163.7)	1.1(0.9,1.2)	502.9(440.4,565.3)	122.1(109.1,135.1)	0.9(0.8,1.0)
Gender						
Female	1469.0(1428.0,1510.0)	456.7(442.4,471.1)	12.5(12.1,12.8)	1212.4(1178.6,1246.2)	376.9(365.1,388.8)	10.3(10.0,10.6)
Male	1856.2(1808.7,1903.6)	515.5(500.1,531.0)	19.2(18.7,19.7)	1532.0(1492.8,1571.1)	425.5(412.7,438.2)	15.8(15.4,16.3)
Age group						
0-19	1385.1(950.0,1820.2)	694.5(443.8,945.2)	0.1(0.0,0.1)	1143.1(784.0,1502.2)	573.2(366.3,780.1)	0.1(0.0,0.1)
20-29	1550.5(988.9,2112.1)	482.8(307.4,658.1)	0.2(0.1,0.3)	1279.6(816.2,1743.1)	398.5(253.7,543.2)	0.2(0.1,0.2)
30-39	1477.3(1254.8,1699.8)	457.0(382.3,531.6)	0.5(0.4,0.6)	1219.2(1035.6,1402.8)	377.1(315.6,438.7)	0.4(0.4,0.5)
40-49	1751.0(1543.8,1958.3)	632.8(547.3,718.3)	2.2(2.0,2.5)	1445.2(1274.1,1616.2)	522.3(451.7,592.8)	1.8(1.6,2.0)
50-59	1607.5(1547.1,1667.8)	526.9(504.3,549.4)	5.3(5.1,5.5)	1326.7(1276.8,1376.5)	434.8(416.2,453.4)	4.4(4.2,4.5)
60-69	1591.4(1535.8,1646.9)	483.6(463.6,503.5)	8.5(8.2,8.8)	1313.4(1267.5,1359.2)	399.1(382.6,415.5)	7.0(6.7,7.2)
70-79	1670.5(1609.8,1731.1)	449.9(433.2,466.7)	9.0(8.6,9.3)	1378.7(1328.6,1428.7)	371.3(357.5,385.1)	7.4(7.1,7.7)
80+	1941.2(1865.7,2016.7)	467.5(448.9,486.0)	6.0(5.7,6.2)	1602.1(1539.8,1664.4)	385.8(370.5,401.1)	4.9(4.7,5.1)
Insurance						
URBMI	1632.2(1573.3,1691.0)	688.6(663.5,713.8)	9.9(9.5,10.2)	1347.0(1298.5,1395.6)	568.3(547.6,589.1)	8.1(7.8,8.4)
UEBMI	1704.9(1666.6,1743.2)	394.8(385.5,404.2)	21.8(21.3,22.3)	1407.1(1375.4,1438.7)	325.9(318.1,333.6)	18.0(17.6,18.4)
Total	1681.6(1649.5,1713.6)	489.0(478.3,499.7)	31.7(31.1,32.3)	1387.8(1361.4,1414.3)	403.6(394.8,412.4)	26.2(25.7,26.7)

Abbreviations: OOP, Out of Pocket. 95% CI in parentheses.

Table S2. Summary of average costs in USD and EUR respectively

	Average cost per visit, USD				Average OOP cost per visit, USD			
	Ischemic	Hemorrhagic	Undetermined	Total	Ischemic	Hemorrhagic	Undetermined	Total
Outpatient	72.1(113.8)	67.5(166.0)	61.0(49.3)	69.6(116.8)	30.5(46.2)	20.5(83.3)	16.3(27.4)	26.7(52.1)
Pharmacies	80.5(45.3)	49.3(64.0)	56.3(69.6)	66.4(56.8)	59.9(35.4)	6.5(17.3)	9.6(20.1)	35.8(39.1)
Primary	52.3(70.6)	68.4(70.8)	49.9(49.9)	53.6(68.4)	20.2(32.7)	20.8(26.1)	11.0(22.3)	19.0(31.0)
Secondary	69.7(83.3)	70.7(89.8)	55.5(35.1)	66.6(76.4)	27.9(38.4)	25.0(26.2)	14.3(17.8)	24.4(33.5)
Tertiary	85.6(159.7)	103.7(348.8)	70.9(56.2)	84.3(171.4)	30.3(56.9)	46.4(183.8)	20.6(34.6)	29.8(73.9)
Inpatient	1655.5(2163.4)	4244.0(6984.0)	1804.0(2727.4)	2007.9(3386.7)	445.7(785.1)	1388.6(3363.8)	478.5(807.1)	573.6(1471.5)
Primary	909.0(1413.0)	1872.1(3768.7)	704.2(1025.3)	989.9(1770.4)	194.3(391.9)	466.7(1421.9)	150.2(225.1)	217.5(568.3)
Secondary	1268.4(1330.0)	2787.8(5040.9)	1701.7(2508.7)	1462.3(2234.3)	337.4(491.5)	854.5(2432.0)	419.7(580.3)	402.2(982.6)
Tertiary	2345.3(2800.2)	5751.0(8207.8)	2328.0(3195.9)	2904.7(4389.1)	655.7(1048.1)	1951.6(4039.0)	660.1(1044.0)	868.7(1956.9)
	Average cost per visit, EUR				Average OOP cost per visit, EUR			
	Ischemic	Hemorrhagic	Undetermined	Total	Ischemic	Hemorrhagic	Undetermined	Total
Outpatient	59.5(93.9)	55.7(137.0)	50.3(40.7)	57.4(96.4)	25.2(38.1)	17.0(68.7)	13.4(22.6)	22.0(43.0)
Pharmacies	66.5(37.4)	40.7(52.8)	46.5(57.5)	54.8(46.8)	49.5(29.3)	5.3(14.3)	7.9(16.6)	29.5(32.3)
Primary	43.1(58.3)	56.5(58.5)	41.2(41.1)	44.2(56.5)	16.7(27.0)	17.1(21.5)	9.1(18.4)	15.7(25.6)
Secondary	57.5(68.7)	58.3(74.1)	45.8(28.9)	55.0(63.1)	23.0(31.7)	20.6(21.6)	11.8(14.7)	20.1(27.6)
Tertiary	70.7(131.8)	85.6(287.9)	58.5(46.3)	69.6(141.4)	25.0(47.0)	38.3(151.7)	17.0(28.6)	24.6(61.0)
Inpatient	1366.3(1785.5)	3502.6(5764.0)	1488.8(2251.0)	1657.2(2795.1)	367.8(647.9)	1146.0(2776.2)	394.9(666.1)	473.4(1214.5)
Primary	750.2(1166.2)	1545.0(3110.4)	581.2(846.2)	817.0(1461.1)	160.4(323.4)	385.2(1173.5)	123.9(185.8)	179.5(469.0)
Secondary	1046.8(1097.7)	2300.8(4160.3)	1404.4(2070.5)	1206.8(1844.0)	278.5(405.7)	705.2(2007.2)	346.4(478.9)	331.9(810.9)
Tertiary	1935.6(2311.0)	4746.4(6774.0)	1921.4(2637.6)	2397.3(3622.4)	541.1(865.0)	1610.7(3333.5)	544.8(861.6)	717.0(1615.0)

Standard deviation in parentheses.

Table S3. Results of the multivariate analyses

	Outpatient			Inpatient		
	The average number of annual visits	Average cost per visit	Average OOP cost per visit	The average number of annual admission	Average cost per admission	Average OOP cost per admission
Pathological type						
Hemorrhagic	-0.003(0.001) ***	-0.059(0.001) ***	-0.390(0.001) ***	-0.063(0.001) ***	0.962(0.001) ***	1.093(0.001) ***
Undetermined	0.338(0.001) ***	-0.170(0.001) ***	-0.572(0.001) ***	-1.220(0.002) ***	0.090(0.002) ***	0.076(0.003) ***
Gender						
Male	0.089(0.000) ***	-0.002(0.001) **	0.063(0.001) ***	0.185(0.001) ***	0.034(0.001) ***	0.008(0.001) ***
Age group						
20-29	-0.257(0.007) ***	-0.114(0.010) ***	-0.117(0.016) ***	-0.480(0.008) ***	0.849(0.011) ***	0.494(0.015) ***
30-39	-0.289(0.006) ***	-0.082(0.010) ***	-0.112(0.015) ***	0.049(0.007) ***	0.315(0.009) ***	0.201(0.012) ***
40-49	-0.081(0.006) ***	0.251(0.010) ***	0.093(0.015) ***	0.339(0.006) ***	0.223(0.008) ***	0.166(0.012) ***
50-59	0.151(0.006) ***	0.420(0.010) ***	0.178(0.015) ***	0.418(0.006) ***	0.151(0.008) ***	0.056(0.012) ***
60-69	0.172(0.006) ***	0.444(0.010) ***	0.170(0.015) ***	0.483(0.006) ***	0.166(0.008) ***	-0.014(0.012)
70-79	0.137(0.006) ***	0.457(0.010) ***	0.144(0.015) ***	0.538(0.006) ***	0.222(0.008) ***	-0.031(0.012) **
80+	0.040(0.006) ***	0.428(0.010) ***	0.083(0.015) ***	0.574(0.006) ***	0.378(0.008) ***	-0.007(0.012)
Insurance						
UEBMI	0.881(0.001) ***	0.522(0.001) ***	0.163(0.001) ***	-0.283(0.001) ***	0.288(0.001) ***	-0.349(0.001) ***
Year						
2014.	-0.038(0.001) ***	-0.169(0.001) ***	0.336(0.002) ***	-0.138(0.001) ***	0.030(0.001) ***	0.062(0.002) ***
2015.	0.397(0.001) ***	-0.091(0.001) ***	0.340(0.002) ***	-0.102(0.001) ***	0.087(0.001) ***	0.044(0.001) ***
2016.	0.450(0.001) ***	-0.119(0.001) ***	0.403(0.002) ***	-0.050(0.001) ***	0.123(0.001) ***	0.102(0.001) ***
Constant	-0.652(0.006) ***	5.342(0.009) ***	4.614(0.015) ***	-0.482(0.006) ***	8.749(0.008) ***	8.076(0.012) ***

Standard errors in parentheses. *** P<.001, ** P<.01.

Table S4 Medical cost associated with sociodemographic characteristics, pathological type and year in USD and EUR respectively

	Outpatient (USD)		Inpatient (USD)	
	Average cost per visit	Average OOP cost per visit	Average cost per admission	Average OOP cost per admission
**Baseline**	33.014(32.400,33.628)***	15.942(15.481,16.404)***	996.634(980.500,1012.767)***	508.280(496.776,519.784)***
**Increment**				
Pathological type				
Hemorrhagic	-4.137(-4.261,-4.013)***	-9.773(-9.836,-9.710)***	2667.429(2659.224,2675.634)***	889.516(885.991,893.041)***
Undetermined	-11.248(-11.361,-11.136)***	-13.174(-13.231,-13.117)***	154.650(145.909,163.391)***	35.429(32.153,38.705)***
Gender				
Male	-0.131(-0.223,-0.040)**	1.660(1.606,1.714)***	68.134(65.309,70.959)***	4.740(3.601,5.879)***
Age group				
20-29	-4.910(-5.820,-3.999)***	-2.543(-3.259,-1.827)***	2143.280(2086.247,2200.312)***	360.147(338.491,381.803)***
30-39	-3.578(-4.453,-2.704)***	-2.453(-3.140,-1.767)***	592.095(563.452,620.738)***	125.441(111.611,139.271)***
40-49	13.028(12.165,13.891)***	2.260(1.584,2.935)***	399.713(373.382,426.043)***	101.576(88.639,114.513)***
50-59	23.818(22.963,24.674)***	4.481(3.811,5.152)***	262.021(236.062,287.980)***	32.214(19.450,44.978)***
60-69	25.512(24.659,26.365)***	4.263(3.594,4.933)***	288.690(262.786,314.593)***	-7.720(-20.453,5.012)
70-79	26.462(25.609,27.315)***	3.581(2.911,4.250)***	397.358(371.425,423.290)***	-17.320(-30.057,-4.584)**
80+	24.398(23.542,25.255)***	2.008(1.337,2.678)***	735.387(709.277,761.498)***	-3.836(-16.603,8.931)
Insurance				
UEBMI	30.093(30.002,30.183)***	4.083(4.015,4.151)***	558.271(555.531,561.010)***	-205.744(-207.009,-204.480)***
Year				
2014.	-11.965(-12.129,-11.801)***	7.695(7.613,7.777)***	57.203(53.146,61.261)***	34.816(33.150,36.482)***
2015.	-6.671(-6.823,-6.518)***	7.796(7.726,7.866)***	169.755(165.884,173.625)***	24.545(23.013,26.077)***
2016.	-8.657(-8.807,-8.507)***	9.568(9.497,9.638)***	244.763(240.948,248.577)***	58.144(56.618,59.669)***
	Outpatient (EUR)		Inpatient (EUR)	
	Average cost per visit	Average OOP cost per visit	Average cost per admission	Average OOP cost per admission

**Baseline**	27.247(26.740,27.754)***	13.158(12.777,13.539)***	822.538(809.223,835.853)***	419.491(409.997,428.986)***
**Increment**				
Pathological type				
Hemorrhagic	-3.414(-3.517,-3.312)***	-8.066(-8.118,-8.014)***	2201.472(2194.701,2208.244)***	734.132(731.222,737.041)***
Undetermined	-9.283(-9.376,-9.191)***	-10.873(-10.920,-10.825)***	127.635(120.421,134.850)***	29.240(26.536,31.944)***
Gender				
Male	-0.108(-0.184,-0.033)**	1.370(1.325,1.415)***	56.232(53.900,58.563)***	3.912(2.972,4.852)***
Age group				
20-29	-4.052(-4.804,-3.300)***	-2.099(-2.690,-1.508)***	1768.883(1721.814,1815.953)***	297.235(279.362,315.108)***
30-39	-2.953(-3.675,-2.232)***	-2.025(-2.591,-1.458)***	488.665(465.026,512.305)***	103.529(92.114,114.943)***
40-49	10.752(10.040,11.465)***	1.865(1.307,2.423)***	329.889(308.158,351.620)***	83.832(73.156,94.509)***
50-59	19.658(18.952,20.364)***	3.699(3.145,4.252)***	216.250(194.826,237.675)***	26.587(16.052,37.121)***
60-69	21.055(20.351,21.759)***	3.518(2.966,4.071)***	238.260(216.882,259.639)***	-6.372(-16.880,4.137)
70-79	21.840(21.136,22.544)***	2.955(2.403,3.508)***	327.946(306.543,349.348)***	-14.295(-24.806,-3.783)**
80+	20.136(19.430,20.843)***	1.657(1.104,2.210)***	606.927(585.378,628.476)***	-3.166(-13.703,7.371)
Insurance				
UEBMI	24.836(24.761,24.911)***	3.370(3.314,3.426)***	460.750(458.489,463.011)***	-169.804(-170.847,-168.760)***
Year				
2014.	-9.875(-10.010,-9.739)***	6.351(6.283,6.418)***	47.211(43.862,50.559)***	28.734(27.360,30.109)***
2015.	-5.505(-5.631,-5.379)***	6.434(6.376,6.492)***	140.101(136.907,143.295)***	20.257(18.993,21.522)***
2016.	-7.145(-7.269,-7.021)***	7.896(7.838,7.955)***	202.006(198.858,205.155)***	47.987(46.728,49.246)***

The baseline represents the utilization and cost for an under 18-year-old female with Urban Residents' Basic Medical Insurance and ischemic stroke in 2013. 95% CI in parentheses. *** *P*<.001, ** *P*<.01.

.
